# Annotations

**Published:** 1889-05-18

**Authors:** 


### Annotations.

**"Cuckoo!"** To be wakened by the call of the cuckoo close to your bed-room window at five o'clock on a May morning is decidedly a novel sensation to a town man, and as welcome as it is novel. It contrasts not unfavourably with another sound which must be familiar to many dwellers in suburban London—the sound of the "re-distribution" of milk-cans by some belated milkman, who selects that portion of the street or square fronting your own house for duties which presumably should have been performed at the dairy. Highly nervous persons have a perfect horror of certain street sounds. The barrel organ is the "diabolus" of some and the crowing cock of others; but not a few lose both sleep and temper by the daily rattling which accompanies the rearranging of a cartload of milk-cans. A still greater contrast to the call of the cuckoo outside your bed-room window at five a.m. is the sight, to say nothing of the sounds, of London Bridge at ten. The crowd is great, some-times dense; the procession of heavy waggons on each side; the hansoms running tilt against the "busses" in the middle, and the "busses" steadily lumbering across regardless of difficulties and interruptions; the continuous flow of foot passengers into the City; the innumerable vessels of all sorts lying below the bridge, and the rush of the trains as they cross the river above—all these together make a scene of striking and ordered confusion hardly to be witnessed elsewhere. The bridge is an epitome of London life, as the cry of the cuckoo is of life in the country. The sudden rush of a kind of spring-summer upon us reminds us that "all work and no play" will not do for town dwellers in hot weather. If any "sky" prophet could tell us what kind of summer we are going to have we might at once prepare for it; and, if it is to be fine, enjoy it from the first to the last day. Is it to be hot like 1887? If so, we must look out, if not for "a lodge in some vast wilderness," at least, for a cool, hilly retreat where a calico tent may be erected near "some boundless contiguity of shade." We counsel our London *confreres* of all sorts to think of both health and pleasure. The eight months of bad weather which we "enjoy" in this climate are quite sufficient for work of a highly strenuous kind. In summer the tension of the brain must be relaxed, and the "loins of the mind," as St. Paul has it, must be ungirt. There is a time for all things, and sunshine and blue skies are Nature's invitation cards to the hard-worked man of the town, bidding him lay down his tools, put on his straw hat and come out and enjoy himself.

**A Magisterial Valuation of Child-Life.** Every accused person should have a fair hearing, and William Humphrey, labourer, of Pulborough, with Sarah his wife, appear to have had one. Whether they have received a just sentence is much more open to question. According to the current number of the *Child's Guardian*, these persons have recently lost two children by death, and a third child five years old has only been saved from a similar fate by the intervention of outside persons. The rector of the parish, his wife, the curate, the doctor, and a neighbour, all gave evidence. Their evidence leaves little room for doubt that the two dead children were starved to death, and that the one which was rescued was within sight of death from the same cause. William Humphrey, it appears, is a labourer, and his wages amount to 11s. a week, with a house rent free. Not very much, truly, on which to maintain a wife and three children. Still, it must be remarked that thousands of labourers live on similar wages, and that, moreover, Humphrey's wife might have added to the family income by field or other work. Drink, and not small wages, is stated to have been the cause of the Humphreys' shocking and unnatural crime. The police-sergeant, Maynard, said he knew the defendants drank. The husband had been five times convicted of drunkenness. The mother of these children, born and reared under the shadow of the Christian Church, was in the habit of leaving them, for many hours together, alone in the house. The youngest, which was a child at breast, was often thus left. During the month of February the mother of this helpless infant on several occasions left her house at seven o'clock in the morning, and did not return until four in the afternoon. A child at breast deliberately left by a human mother for nine hours without food! How many murderers have displayed such slow, stony-hearted, incredible cruelty as that? On the 17th February one of the children died, on the 20th a second. There seems to have been but one left, and it a mere infant of five years old. On the day of the second child's death the rector went to the house in the afternoon. He found the door open and a cold east wind blowing in. Little Fanny, aged five, was lying on some sacks, on a board, apparently in the last stage of consumption. At the other end of the same board lay the dead body of another child, absolutely naked. Upstairs the rector found a second dead child in a room, whose single article of furniture was the coffin in which the little one was placed. What pitying angel saw and recorded the pains and hunger pangs of those three children as they slowly starved to death in the cold months of winter in that foodless and fireless home? What thought the Christian Pulborough magistrates of the same pains and hunger pangs? For the magistrates of Pulborough will almost certainly "profess and call themselves *Christians!*" They thought a month's imprisonment with hard labour an adequate measure of the sufferings of those three helpless little ones and of the value of their poor lives. If Humphrey had been a poacher, would they have valued a hare or a pheasant at the same rate? Truly we have much need, not only of a doctrine, but of a practice of "development" in these "Christian," "cultured," and "scientific" times.

**Carlsbad.** The Carlsbad season has commenced. From May to October the fashionable German watering-place will be crowded with visitors of every nationality seeking to be cured of every disease under the sun, from chronic *ennui* upwards, by drinking its famous mineral waters. As the springs do not cease to flow in the autumn, many people who feel themselves benefited by them remain at Carlsbad throughout the winter. There are no fewer than seventeen mineral springs, but the chief of these is the Sprudel, a self-willed spring, that has a bad habit of spurting up in unexpected quarters, without regard to the possible damage it may thereby do. A very cantankerous spring in this Sprudel. Nature has provided it with a large supply of carbonic acid gas, which, whenever it manages to overcome the pressure of the column of water which confines it, sends it out in jets. If the stream finds itself obstructed in any way, even by the earthy ingredients it has deposited in its course, it flies up, forcing its way through the solid earth above. The chief mineral constituents of the Sprudel water are soda in various combinations, with a little lithia and potash, and it is best adapted for the relief of affections of the stomach and abdominal organs. At first the springs were used only as baths, and the washings of Naaman the Syrian were nothing to those endured by conscientious visitors to Carlsbad. The proper thing was to remain in the bath till the skin became sore, and the remedy became known as the "Hautfresskur," or flaying-bath. This method went out of fashion about the middle of last century, and then came a time of excessive drinking of the water. Now it is used in both ways, but in moderation. Of course the mineral water alone does not effect the cure; it is aided by clear fresh air, fine scenery, pleasant company, and plenty of amusement. But it is evident that the value of Carlsbad does not lie merely in fashion or fancy, or it would not have preserved its popularity during more than three centuries. Its mineral springs were known in the twelfth century, and in the sixteenth it was a popular bathing-place. Since that time it has been visited by many notable persons, among whom we may name Peter the Great; Schiller, who spent his honeymoon there; and Goethe, who went there for fourteen seasons.

